# mechanistic Characterization of the nuclear import and export signals of VZV ORF9

**Published:** 2011-01-10

**Authors:** Mingsheng Cai, Shuai Wang, Chunfu Zheng

**Affiliations:** 1 State Key Laboratory of Virology, Wuhan Institute of Virology, Chinese Academy of Sciences, Wuhan 430071, PR China

## 

The ORF9 protein, a VZV-encoded late protein consisting of 302 amino acid (aa) residues, is a member of the highly conserved alphaherpesvirus UL49 gene family but shares limited identity with the UL49 prototype, HSV-1 VP22. As the orthologue of HSV-1 VP22, ORF9 is believed to be a major constituent of the VZV virion tegument. HSV-1 VP22 has been extensively studied; however, the functional properties of ORF9 are less well understood, despite the fact that its transcript is the most abundant viral message expressed during VZV lytic infection. As an important step toward understanding the detailed functions of ORF9 *in vivo* is to determine its precise subcellular localization, in the work presented here, to avoid the flaw of fixation protocol, living cells fluorescence microscopy technique, which is widely applied and developed in our group, was applied to deeply analyze the intracellular distribution of ORF9 protein and, to characterize its functional nuclear localization signal (NLS) and nuclear export signal (NES), as well as its transport mechanism in living cells.

Transient expression of ORF9 fused to enhanced yellow fluorescent protein (EYFP) in COS-7 cells showed the predominantly cytoplasmic localization in the absence of other viral proteins. Time-lapse examination of ORF9-EYFP demonstrated that the ORF9 was found to possess a pronounced cytoplasmic stage early post transfection, followed by translocation to the nucleus at a later time, which was consistent with the fact that VP22 is predominantly localized in the cytoplasm early in infection and accumulates in the nucleus late in infection, and the nuclear targeting of VP22 is independent of other viral factors.

By sequence analysis and constructing a series of deletion derivatives of ORF9 fused to EYFP and fluorescence microscopy analysis in live cells, a bona fide bipartite NLS of ORF9 was for the first time determined and mapped to amino acids (aa) 16 to 32 (RRKTTPSYSGQYRTARR), which containing two arginine-rich motifs RRK and RTARR.

Additionally, the NES was identified to locate between the leucine-rich region at amino acids 103 to 117 (LRHELVEDAVYENPL). Besides, ORF9 protein was demonstrated to target to the cytoplasm through the functional NES by Ran and chromosomal region maintenance 1 (CRM1)-dependent pathway, and to the nucleus through importin β-dependent mechanism that does not require importin α5.

